# NFX1-LIKE2 (NFXL2) Suppresses Abscisic Acid Accumulation and Stomatal Closure in *Arabidopsis thaliana*

**DOI:** 10.1371/journal.pone.0026982

**Published:** 2011-11-03

**Authors:** Janina Lisso, Florian Schröder, Joachim Fisahn, Carsten Müssig

**Affiliations:** 1 University of Potsdam, Max Planck Institute of Molecular Plant Physiology, Potsdam-Golm, Germany; 2 Max Planck Institute of Molecular Plant Physiology, Potsdam-Golm, Germany; Instituto de Biología Molecular y Celular de Plantas, Spain

## Abstract

The *NFX1-LIKE1* (*NFXL1*) and *NFXL2* genes were identified as regulators of salt stress responses. The NFXL1 protein is a nuclear factor that positively affects adaptation to salt stress. The *nfxl1-1* loss-of-function mutant displayed reduced survival rates under salt and high light stress. In contrast, the *nfxl2-1* mutant, defective in the *NFXL2* gene, and *NFXL2*-antisense plants exhibited enhanced survival under these conditions. We show here that the loss of *NFXL2* function results in abscisic acid (ABA) overaccumulation, reduced stomatal conductance, and enhanced survival under drought stress. The *nfxl2-1* mutant displayed reduced stomatal aperture under all conditions tested. Fusicoccin treatment, exposition to increasing light intensities, and supply of decreasing CO_2_ concentrations demonstrated full opening capacity of *nfxl2-1* stomata. Reduced stomatal opening presumably is a consequence of elevated ABA levels. Furthermore, seedling growth, root growth, and stomatal closure were hypersensitive to exogenous ABA. The enhanced ABA responses may contribute to the improved drought stress resistance of the mutant. Three *NFXL2* splice variants were cloned and named *NFXL2-78*, *NFXL2-97*, and *NFXL2-100* according to the molecular weight of the putative proteins. Translational fusions to the green fluorescent protein suggest nuclear localisation of the NFXL2 proteins. Stable expression of the *NFXL2-78* splice variant in *nfxl2-1* plants largely complemented the mutant phenotype. Our data show that *NFXL2* controls ABA levels and suppresses ABA responses. *NFXL2* may prevent unnecessary and costly stress adaptation under favourable conditions.

## Introduction

ABA plays a major role in seed maturation, control of germination, and other phases of plant development, but perhaps the most important function of ABA is to regulate plant water balance and osmotic stress tolerance. ABA-deficient mutants and ABA-insensitive mutants readily wilt under drought stress. The role of ABA under drought stress is at least twofold: ABA maintains the water balance mainly through guard cell regulation, and ABA induces genes that confer dehydration tolerance [1].

Significant progress has been made in understanding the signal transduction processes linking the hormone to target responses [2], [3]. The PYRABACTIN RESISTANCE1 (PYR1) and PYR1-LIKE (PYL) receptor family was recently shown to bind ABA and to inhibit activity of protein Ser/Thr phosphatases type 2C (PP2Cs) functioning as negative regulators [4]–[7]. Inhibition of the PP2Cs allows phosphorylation and activation of SnRK2 protein kinases [8]–[12], which in turn may activate transcription factors [13] and ion channels [14]–[17]. This central signalling module represents the earliest events of ABA signal transduction. Further physiological responses to ABA under water stress conditions are largely brought about by changes in gene expression. Transcription factors of various families (e.g., bZIP, B3, HD-ZIP, NAC, WRKY, bHLH, and Zn-finger classes) control genomic ABA responses [18]. The AREB/ABF (ABA-responsive element-binding protein or ABA-responsive element-binding factor) bZIP transcription factors such as AREB1/ABF2, AREB2/ABF4, and ABF3 are key regulators of ABA signalling in response to osmotic stress [13], [19]–[23]. The AREB/ABF proteins regulate ABRE-dependent gene expression during osmotic stress conditions. Transcription factors of the DREB/CBF (dehydration-response element-binding or C-repeat binding factor) family physically interact with AREB/ABF family transcription factors and control ABA sensitivity [24]. Members of other transcription factor classes have been reported to be both positive and negative regulators of ABA-responsive gene expression [18].

Under drought and salt stress, the endogenous ABA level increases. ABA biosynthesis is regulated at several steps and coordinated with the upstream metabolism [1], [25]. When dehydrated plants are rehydrated, the ABA level decreases rapidly.

The changes in gene expression patterns [18], metabolism [26], [27], and physiology [28], [29] in response to ABA, osmotic and salt stress have been intensely analyzed. Little information is available concerning the upstream sensing and signalling that determines ABA accumulation under abiotic stress [30]. The reduction of turgor may be sensed as a mechanical change that stimulates ABA accumulation. Protein kinases (e.g. histidine sensor kinases, mitogen-activated protein kinases [MAPKs], calcium-dependent protein kinases [CDPKs], and SnRK2s) may play important roles.

In this study, we characterize the Arabidopsis gene *NFX1-LIKE2* (*NFXL2*). *NFXL2* was identified as homologue of the *NFXL1* gene [31]. NFXL proteins are found in animals, fungi, plants, and protists, and may be ubiquitous in eukaryotes [32]. The human NFX1 protein constituted the protein family [33]–[35]. Unique structural features of the NFX1 and NFXL proteins are the Cys-rich region and the specific RING finger motif. The Cys-rich region frequently comprises more than 500 amino acids and harbors several NFX1-type zinc finger domains [32]. The pattern C–X(1–6)-H-X-C-X3-C(H/C)-X(3–4)-(H/C)-X(1–10)-C describes the NFX1-type zinc finger (X can be any amino acids; two positions can be either His or Cys). The Cys-rich region is furthermore characterized by additional highly conserved residues (predominantly Cys residues). The Cys-rich region of the human NFX1 protein is required for binding to specific promoter elements [33], [34]. The N-termini of the Cys-rich regions usually are identical between isoforms, but the C-termini vary. The structure and complexity of the Cys-rich region may represent a major determinant of protein function. The whole Cys-rich region rather than individual NFX1-type zinc finger motifs may specify binding properties.

The second unique feature of the NFX1 and NFXL proteins is a specific RING finger motif. The RING finger is characterized by a C_4_HC_3_ Zn ligand signature and additional conserved amino acids [32]. E3 ubiquitin ligase activity is an intrinsic function of many RING finger proteins, and NFX1-like proteins may also have E3 activity [36]. A number of E3s have been identified that control ABA-biosynthesis and ABA responses [37]. For example, the RING-type E3 ligase SDIR1 is involved in ABA-related stress signal transduction [38], and the RING-H2 domain-containing XERICO protein controls ABA biosynthesis [39]. RING domain-containing proteins also function as part of multi-subunit E3 complexes such as SKP-Cullin-F-box (SCF) complexes. For example, the F-box protein DOR serves as negative regulator of ABA-induced stomatal closure under drought stress [40].

Plants use the ubiquitin proteasome system to alter their proteome to mediate changes required for responses to abiotic stress [41], [42]. However, ubiquitination is a versatile post-translational modification that is not only used for targeting to the proteasome. For example, monoubiquitination can control nuclear localization and transcriptional activity of transcription factors [43]. Further nuclear proteins such as histones and RNA polymerase II are subject to ubiquitination [44]. The functional relevance of the putative E3 ligase activity of the NFX1 and NFXL proteins is unknown.

In this study, we functionally characterize the Arabidopsis *NFXL2* gene. The loss of *NFXL2* resulted in elevated ABA-levels, reduced stomatal aperture, and enhanced survival of water stress. *NFXL2* may prevent unnecessary and costly stress adaptation under favourable conditions. Deactivation of NFXL2 action may be inevitable for proper stress responses.

## Results

### Structure and subcellular localisation of NFXL2 proteins

**Figure 1:**
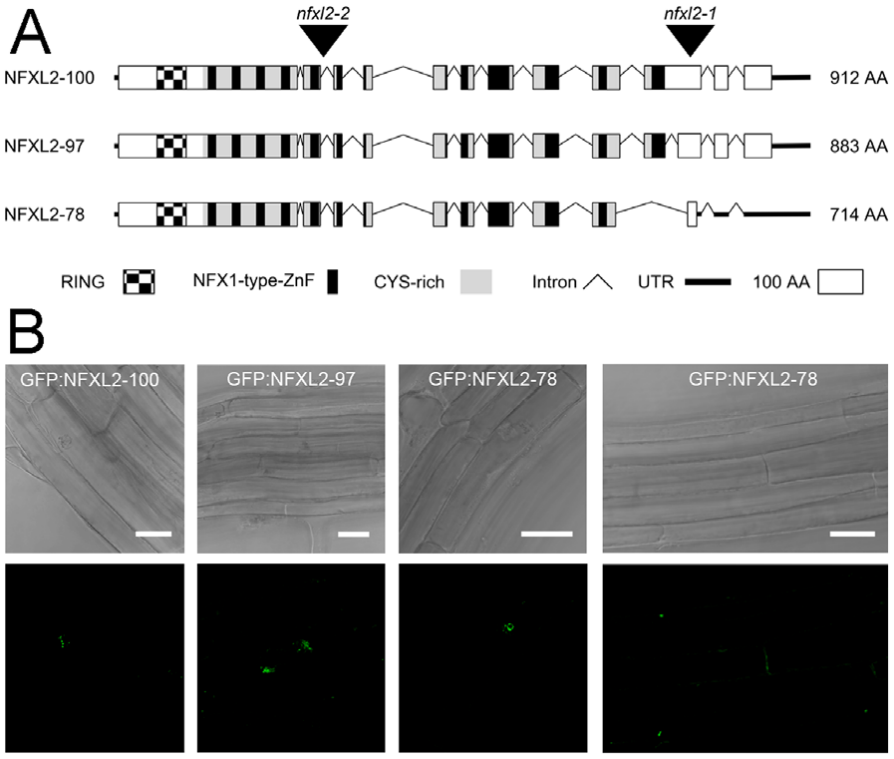
Structure and subcellular localization of NFXL2 proteins. A. Exon-intron structure and domain organisation of NFXL2 proteins. Exons are represented by boxes, untranslated regions by thick lines, and introns by thin lines. T-DNA insertions in the *nfxl2-1* (SALK_140301) and *nfxl2-2* (GABI-Kat line 668B09) mutants are indicated. B. Subcellular localisation of GFP:NFXL2-100, GFP:NFXL2-97, and GFP:NFXL2-78 proteins. GFP-fluorescent images and bright field images of roots of stably transformed Arabidopsis plants. The scale bar represents 25 µm.

Screening of RACE-libraries resulted in the identification of three *NFXL2* splice variants termed *NFXL2-78*, *NFXL2-97*, and *NFXL2-100* according to the putative molecular weight of the encoded proteins. The NFXL2-97 isoform differs only slightly in the C-terminus from the previously identified NFXL2-100 isoform. An 87 bp intron corresponding to amino acids 732 to 760 of the NFXL2-100 protein is removed in the NFXL2-97 isoform (Figure 1A). The N-terminal primary structure of the NFXL2-78 isoform (comprising 697 amino acids) is identical to the NFXL2-97 and NFXL2-100 isoforms, but alternative donor and acceptor sites account for sequence divergence of the C-termini (Figure 1A). The three putative NFXL2 proteins show the characteristic features of NFXL proteins (Figure 1A), namely the N-terminal C_4_HC_3_ RING-finger motif and the Cys-rich region. The length of the Cys-rich region of the NFXL2-78 isoform is reduced in comparison to the NFXL2-97 and NFXL2-100 isoforms (Figure 1A). The NFXL2-78 protein comprises 10 NFX1-type zinc finger motifs, the NFXL2-97 and NFXL2-100 isoforms comprise 11 NFX1-type zinc finger motifs.

To determine whether NFXL2-78 is imported into the nucleus, a GFP (green fluorescent protein)-tagged NFXL2-78 protein was stably expressed in Arabidopsis plants. Green fluorescence was detectable in the nuclei of root cells (Figure 1B). Thus, the NFXL2-78 protein directs GFP to the nucleus. Analysis of plants carrying the 35S::GFP:NFXL2-97 and 35S::GFP:NFXL2-100 constructs suggests that the NFXL2-97 and NFXL2-100 isoforms presumably are also nuclear proteins.

### *NFXL2* expression pattern

**Figure 2:**
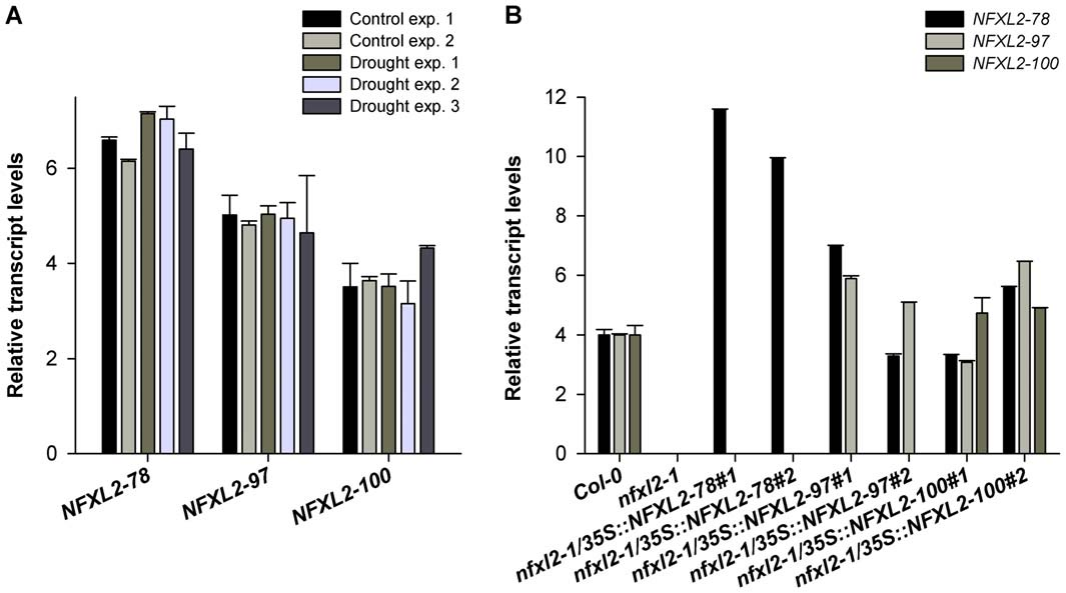
*NFXL2* transcript levels. A. Real-time RT-PCR analysis of *NFXL2* transcript levels in shoots of wild-type (Col-0) plants. For drought treatment, four-week-old soil-grown plants were withheld from water for 3 d. Higher bars indicate higher transcript levels. A difference of one indicates a fold change of two. Error bars: SE of three technical replicates. B. Real-time RT-PCR analysis of *NFXL2* transcript levels in shoots of four-week-old *nfxl2-1*/35S::NFXL2-78, *nfxl2-1*/35S::NFXL2-97, and *nfxl2-1*/35S::NFXL2-100 plants in comparison to the wild type and *nfxl2-1* mutant. Wild-type transcript levels were arbitrarily adjusted to a relative value (i.e. 4). The *nfxl2-1* mutant is not able to produce the *NFXL2-*78/*97*/*100* mRNAs. Transcript levels of the transgenic lines were given relative to the wild type. Higher bars indicate higher transcript levels. A difference of one indicates a fold change of two. Error bars: SE of three technical replicates.

Web-based platforms such as Genevestigator [45] and the Arabidopsis eFP Browser [46] indicate the presence of relatively steady levels of *NFXL2* transcript in most tissues and cell types. Transcript levels barely change under biotic and abiotic stress conditions, upon treatment with elicitors and other chemicals, and under different light and nutrient conditions. The *NFXL2* gene is comparably expressed in mesophyll cells and guard cells [47], and is not controlled by ABA and other phytohormones [45]–[47]. Since common expression profiling platforms (i.e., the Affymetrix ATH1 and Affymetrix Tiling arrays) do not discriminate between the *NFXL2* splice variants, *NFXL2-78*, *NFXL2-97*, and *NFXL2-100* transcript levels were determined in shoot material of well-watered and drought-stressed plants by means of quantitative RT-PCR. Transcript levels were not significantly different under both conditions (Figure 2A).

### Loss of *NFXL2* prevents wilting under drought stress

In a previous study, the *nfxl2-1* loss-of-function mutant and *NFXL2*-antisense lines showed higher survival rates under salt stress/high light conditions in comparison to the wild type [31]. Higher survival rates were accompanied by higher F_v_/F_m_ ratios, indicating less damage to the photosynthetic apparatus. These findings suggested that *NFXL2* suppresses salt stress responses.

The *nfxl2-1* mutant showed only minor phenotypic changes under well-watered conditions. The mutant had a tendency to early flowering (data not shown), the dry weight content was slightly increased (Table S1), but the mutant did not display obvious morphological alterations. An additional T-DNA insertion mutant was identified and termed *nfxl2-2*. The *nfxl2-1* and *nfxl2-2* mutants exhibited similar phenotypes (see below).

**Figure 3:**
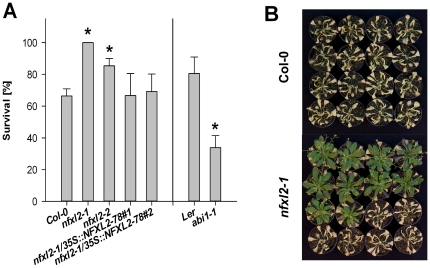
Drought stress tolerance. A. 4-week-old soil-grown plants were withheld from water for 7 d. Survival rates were determined 7 d after water resupply. The number of surviving plants in five independent experiments is represented as the percentage (mean±SE) of the number of total plants (approximately 35 plants per genotype and experiment). Mutant values denoted with an asterisk are significantly different from those of their wild type (t test, P<0.01). B. For drought treatment, 4-week-old soil-grown plants were withheld from water for 9 d. Photos were taken 7 d after water resupply. Plants were arranged according to the visual phenotype. Wild-type plants were not able to survive the prolonged drought period, but approximately 60% of *nfxl2-1* plants were still turgescent and viable.

Survival experiments were performed in order to test for drought stress resistance. Plants were subjected to water withdrawal for seven days. The percentage of surviving plants was determined seven days later after water resupply. Five independent experiments were performed. The *nfxl2-1* and *nfxl2-2* mutants grew significantly better than wild-type plants. All *nfxl2-1* plants survived the drought period; the survival rates of the *nfxl2-2* and wild-type plants accounted for 85 and 66%, respectively (Figure 3A). Water withdrawal for nine days with subsequent rehydration resulted in wilting of all wild-type plants, but approximately 60% of the *nfxl2-1* plants survived and were still turgescent (Figure 3B).

**Figure 4:**
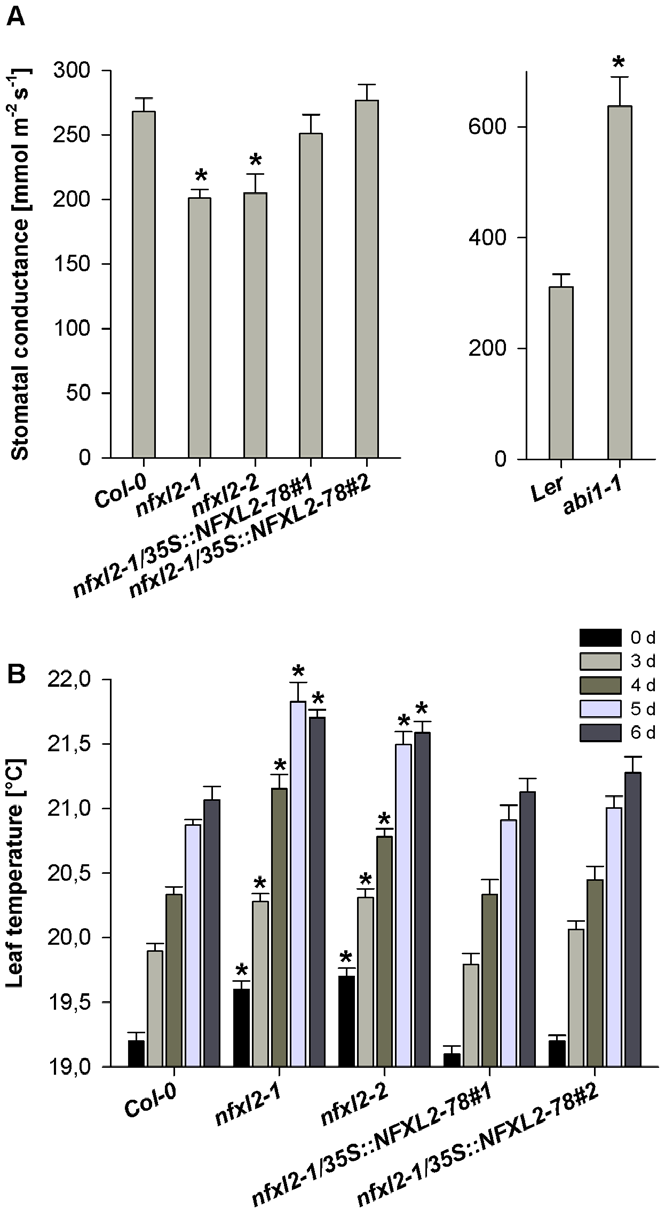
Stomatal conductance and leaf temperature under drought stress. A. Stomatal conductance was determined with a Decagon SC-1 porometer at day 3 of a drought stress experiment. Data are given as mean±SE (n = 10 to 15 leaves per genotype). Mutant values denoted with an asterisk are significantly different from those of their wild type (t test, P<0.001).B. Leaf temperature calculated from the quantification of infrared images (means±SE; n = 10 to 15 leaves per genotype). Values denoted with an asterisk are significantly different from the wild type (t test, P<0.001).

The ABA-insensitive *abi1-1* mutant was used as stress-sensitive control [48]. It exhibited strong wilting and low survival rates (Figure 3A). ABA-induced stomatal closing is impaired in *abi1-1* [49]–[51]. Water vapour flux at day 3 of the drought experiment was clearly elevated (215% in comparison to the wild type [Ler]) (Figure 4A). In contrast, the *nfxl2-1* and *nfxl2-2* mutants showed significantly lower stomatal conductance (75 and 76% of wild type [Col-0], respectively) (Figure 4A). Reduced stomatal conductance was associated with significantly higher leaf temperatures (Figure 4B). These initial findings posed the question whether the NFXL2 proteins control ABA responses.

### Stomatal aperture in *nfxl2-1* leaves is reduced under various environmental conditions

**Figure 5:**
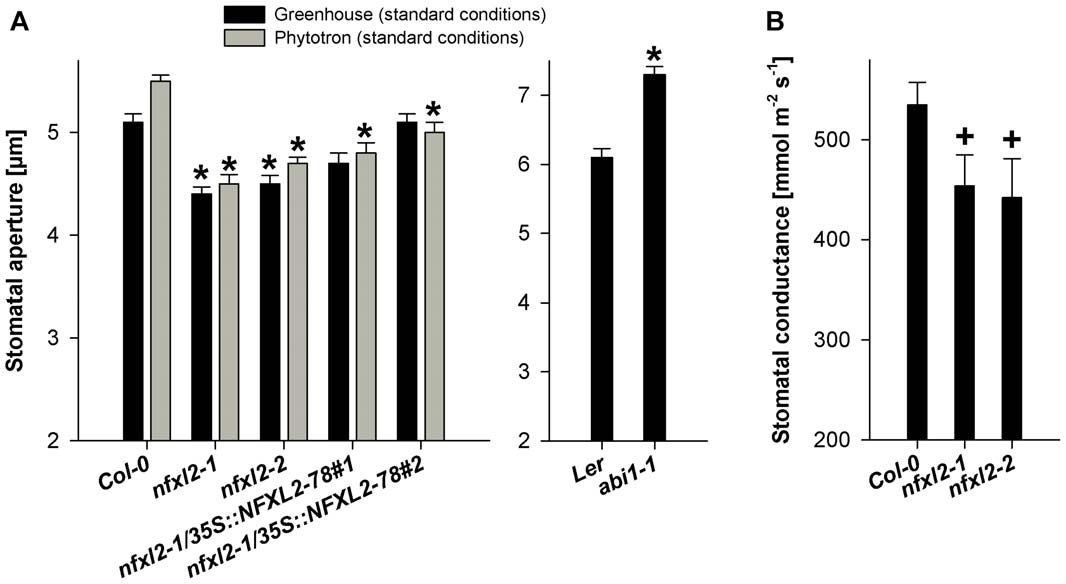
Stomatal aperture and stomatal conductance of well-watered plants grown under long day growth conditions. A. Stomatal aperture was determined from images of silicone rubber imprints of the abaxial surfaces of rosette leaves of five-week-old plants. Plants were grown in a greenhouse under standard conditions (see Methods for details) or in a controlled growth chamber (16 h light, 21°C, 120 µmol m^−2^ s^−1^, 60% humidity; 8 h dark, 19°C, 75% humidity). Data are given as mean±SE of at least 60 stomata per genotype and condition. Values denoted with an asterisk are significantly different from those of their wild type (t test, P<0.001). B. Stomatal conductance of rosette leaves of five-week-old plants was determined with a Decagon SC-1 porometer. Plants were grown in a greenhouse under standard greenhouse conditions. Data are given as mean±SE (n = 10 to 15 leaves per genotype). Mutant values denoted with a positive sign are significantly different from their wild type (t test, P<0.05).

**Figure 6:**
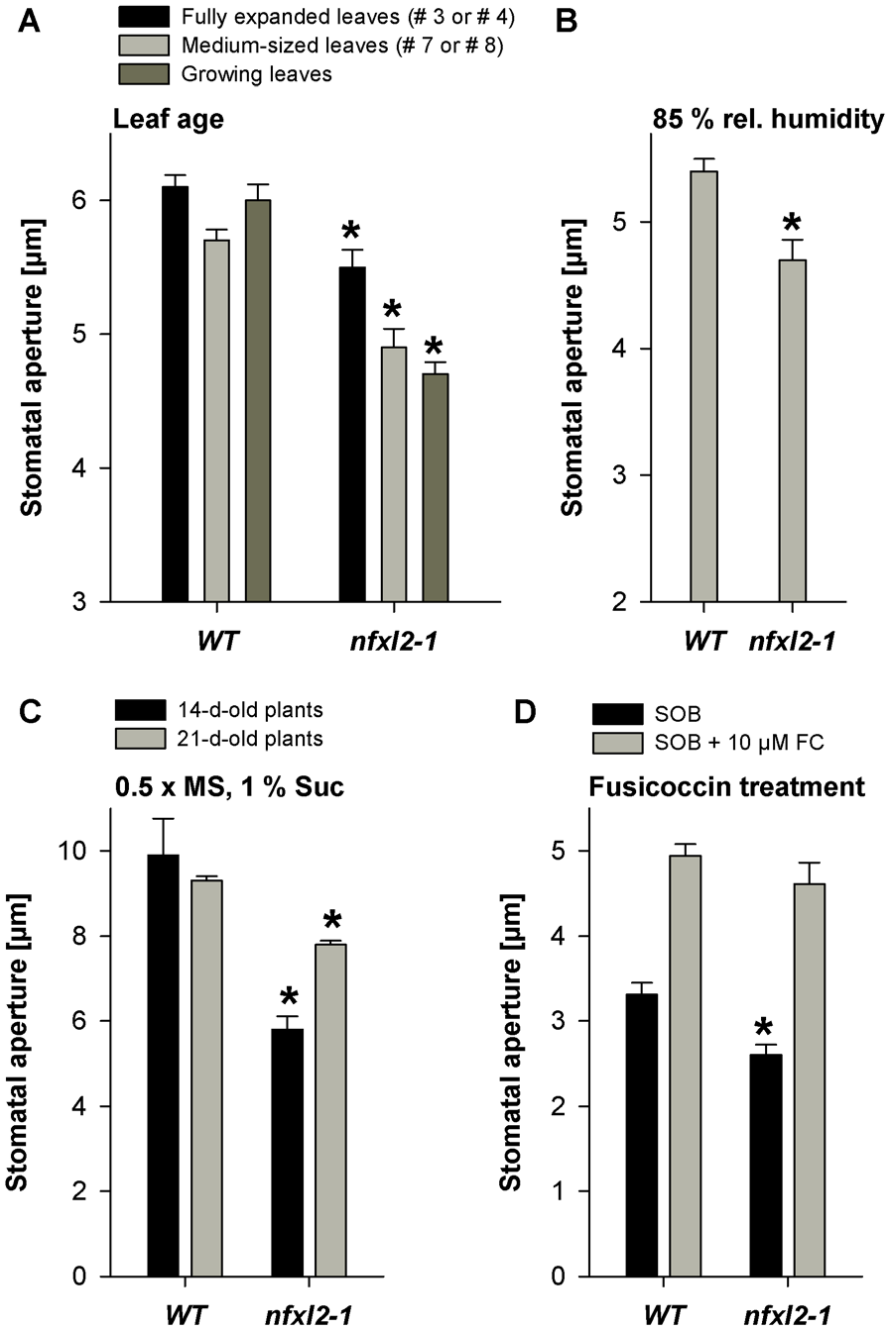
Stomatal aperture of wild-type and *nfxl2-1* plants under different environmental conditions. Stomatal aperture was determined from images of silicone rubber imprints of the abaxial surfaces of rosette leaves (A to C) or determined directly from epidermal peels using a microscope (D). Data are given as mean±SE of at least 60 stomata per genotype and condition. Mutant values denoted with an asterisk are significantly different from their wild type (t test, P<0.001). A. Stomatal aperture of 37-d-old plants grown in soil in a greenhouse under standard conditions. B. Stomatal aperture of 26-d-old wild-type and *nfxl2-1* plants grown in a controlled growth chamber at 85% relative humidity (further climate parameters as given in Figure 5A). C. Stomatal aperture of 14-and 21-d-old plants grown in jars (0.5xMS medium supplemented with 1% sucrose). D. Stomatal response to fusicoccin. Epidermal peels were dissected from rosette leaves of soil-grown plants and incubated for 4 h in darkness either in stomata opening buffer (SOB) or SOB plus 10 µM fusicoccin (SOB+10 µM FC).

Stomatal aperture of well-watered plants was determined microscopically under different environmental conditions. Plants were grown in soil under long day conditions in a greenhouse or in a controlled growth chamber under ambient humidity, or in a controlled growth chamber under high humidity (85%), or in synthetic medium in jars. The *nfxl2-1* mutant displayed significantly smaller stomatal aperture under all conditions (Figure 5A, Figure 6A–C). Reduced stomatal aperture was most pronounced in growing leaves (Figure 6A). In line with the reduced stomatal aperture, stomatal conductance was reduced in plants grown in soil under long day conditions (Figure 5B). Fusicoccin treatments of epidermal peels revealed that *nfxl2-1* stomata were able to open as wide as wild-type stomata (Figure 6D), suggesting that the cellular mechanisms for stomatal opening were intact.

**Figure 7:**
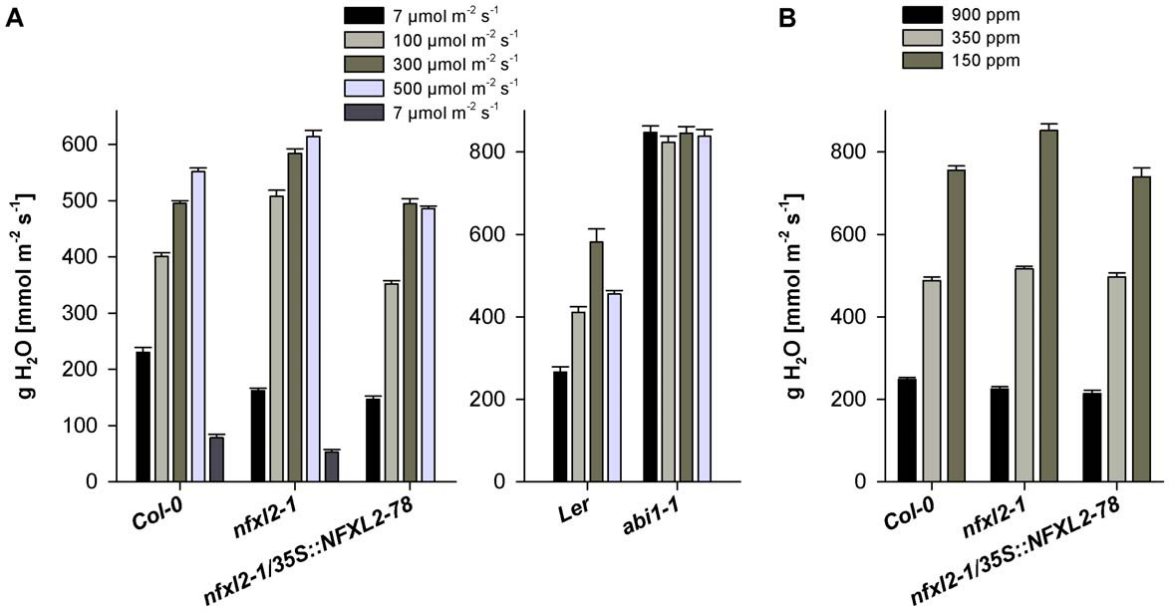
Stomatal conductance in response to light and CO_2_. 4-week-old soil-grown plants were investigated by gas exchange measurements. Graphs represent H_2_O conductance rates obtained during a stepwise change of PFD (A) or CO_2_ concentration (B). Each data point represents the average of 5-10 individual plants±SE.

Light and CO_2_ are key factors in the control of stomatal movement [16], [52]. Low atmospheric CO_2_ concentrations and light induce stomatal opening, high CO_2_ concentrations and darkness cause stomatal closing. In the experiments depicted in Figure 7A, photon flux density (PFD) was increased sequentially from 7 µmol m^−2^ s^−1^ (quasi darkness) to 500 µmol m^−2^ s^−1^. Stomatal conductance of fully expanded rosette leaves of 5-week-old soil-grown plants was determined by infrared gas exchange measurements. The *nfxl2-1* mutant exhibited less transpiration (71% of wild type) at 7 µmol m^−2^ s^−1^, but increasing PFDs caused an elevated stomatal conductance. For example, the increase in PFD from 7 to 500 µmol m^−2^ s^−1^ caused 140% increase of stomatal conductance in the wild type and 287% increase in *nfxl2-1* plants (Figure 7A). Similarly to increasing PFD, a decrease of CO_2_ levels from 900 to 150 ppm resulted in an elevated stomatal conductance in the mutant (Figure 7B). The decrease from 900 to 150 ppm CO_2_ caused 204 and 279% increase of stomatal conductance in wild-type and *nfxl2-1* plants, respectively. These findings indicate that the *nfxl2-1* mutant is fully able to perceive and respond to light and CO_2_. The *nfxl2-1* mutation did not cause a general defect in stomatal functioning, but modified short-term stomatal movements. Although short-term responses to light and CO_2_ were altered, the stomatal aperture under long-term conditions was invariably reduced under all tested conditions (Figure 5A, Figure 6).

### The *nfxl2-1* mutant shows enhanced responses to ABA

**Figure 8:**
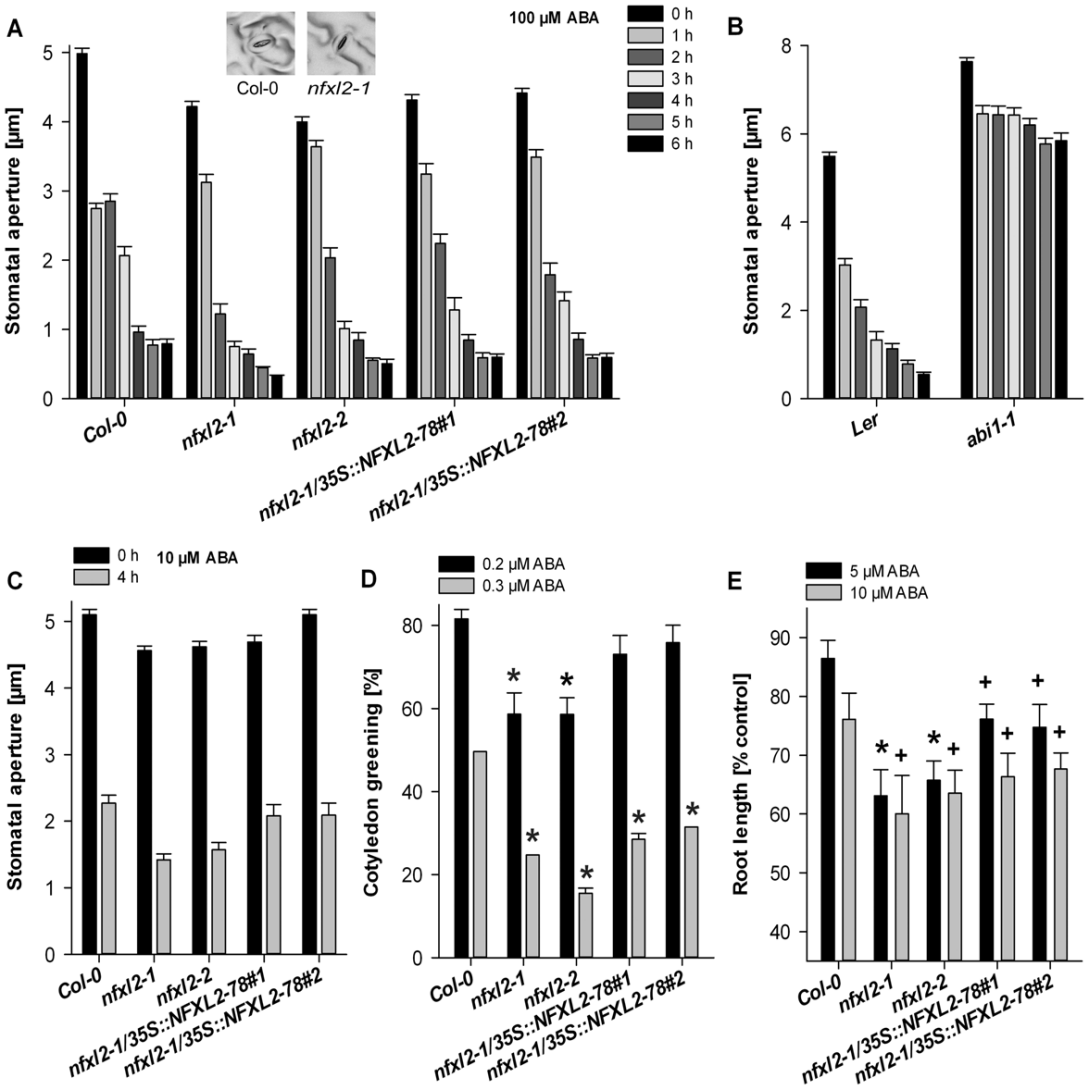
Stomatal aperture in rosette leaves, cotelydon greening, and root growth in the presence of exogenous ABA. A. Stomatal aperture after ABA application. Stomatal aperture was determined from images of silicone rubber imprints of the abaxial surfaces of rosette leaves. Three droplets of an ABA solution (100 µM in SOB, solidified with 0.1% agarose) were supplied to leave petioles. Inset: Imprint of representative wild-type and *nfxl2-1* stomata after 6 h ABA treatment (each photo represents a width of 50 µm).B. Stomatal aperture after ABA application. Three droplets of an ABA solution (100 µM in SOB, solidified with 0.1% agarose) were supplied to leave petioles. Details as in A.C. Stomatal aperture after ABA application. Three droplets of an ABA solution (10 µM in SOB, solidified with 0.1% agarose) were supplied to leave petioles. Further details as in A.D. Cotyledon greening in the presence of exogenous ABA. Seeds were allowed to germinate on half-concentrated MS medium supplemented with 1% sucrose at 4°C. 4-d-old seedlings were transferred to the same medium supplemented with 0.2 and 0.3 µM ABA and cultured at 22°C, with 14 h light (140 µmol m^−2^ s^−1^)/10 h dark photoperiod. After 5 d, the percentage of seedlings with green cotyledons was scored (mean±SE). Values denoted with an asterisk are significantly different from that of their wild type (t test, P<0.001).E. 4-d-old seedlings were transferred to half-concentrated MS medium supplemented with 1% sucrose and 0, 5, and 10 µM ABA. Root length of 14-d-old plants was determined and is given as percentage (mean±SE) of control plants. Values denoted with an * or**+**are significantly different from that of their wild type (t test, P<0.001 or P<0.03).

The effect of exogenous ABA on stomatal aperture was tested by application of synthetic ABA to leaf petioles. Stomata of *nfxl2-1* and *nfxl2-2* mutants exhibited a pronounced response to 100 µM ABA and were nearly fully closed after 6 h treatment (Figure 8A). Percent change (calculated as [[y_2_ – y_1_]/y_1_]*100) of stomatal aperture at the beginning (y_1_: 0 h) and end of treatment (y_2_: 6 h) was higher in both mutants in comparison to the wild type (Col-0: −84%; *nfxl2-1*: −92%; *nfxl2-2*: −87%). The exaggerated ABA-induced stomatal closing may represent the physiological basis for reduced stomatal aperture under various conditions (Figure 5A, Figure 6) and contribute to the enhanced drought tolerance (Figure 3). The *abi1-1* mutant was grown in parallel and barely responded to ABA (Figure 8B). The *nfxl2-1* and *nfxl2-2* mutants also responded stronger to application of 10 µM ABA (percent change after 4 h: Col-0 −56%; *nfxl2-1*: −69%; *nfxl2-2*: −66%) (Figure 8C).

The enhanced response of *nfxl2-1* stomata towards exogenous ABA prompted the analyses of additional ABA responses. First, inhibition of seed germination was tested by supplementation of 0.2 to 1 µM ABA to synthetic medium. ABA dose-response curves of wild-type and *nfxl2-1* plants were similar (data not shown). Thus, the loss of *NFXL2* did not affect ABA-sensitivity of seed germination under the tested conditions. Second, postgerminative growth was tested by scoring cotyledon greening in the presence of ABA. 82 and 50% of wild-type seedlings showed cotyledon greening in the presence of 0.2 and 0.3 µM ABA, respectively, whereas 59 and 25% of the *nfxl2-1* seedlings developed green cotyledons under these conditions (Figure 8D). Third, root elongation was tested in the presence of 5 and 10 µM ABA. The *nfxl2-1* mutant displayed approximately 25% less root growth in the presence of ABA compared with the wild type (Figure 8E). Thus, *NFXL2* suppresses several ABA responses.

### ABA-levels are increased in the *nfxl2-1* mutant

**Figure 9:**
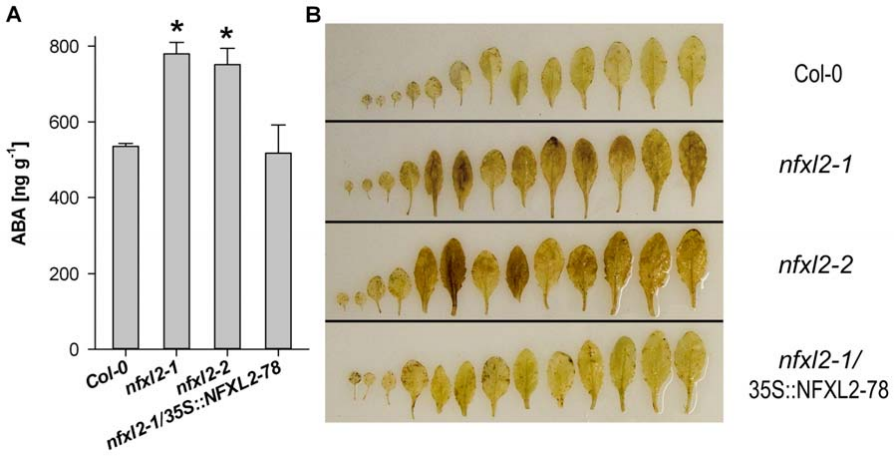
Endogenous ABA levels and imaging of hydrogen peroxide production. A. For drought treatment, soil-grown plants were withheld from water for 3 d. Two independent experiments were analyzed. Results are mean ng ABA per gram fresh weight±SE (n = 3 pools of 10 to 15 plants per genotype and experiment). Values denoted with an * are significantly different from that of their wild type (t test, P<0.001).B. Rosette leaves were infiltrated with 3,3-diamino-benzidine (DAB). Formation of brown polymerisation product indicates H_2_O_2_ formation [75]. Plants were grown in parallel to the plants used in A. Supplemental data are given in Figure S1.

ABA levels were determined in plant material grown in a greenhouse under well-watered or drought conditions. Under standard greenhouse conditions, four-week-old wild-type, *nfxl2-1*, and *nfxl2-2* plants accumulated 29±1, 33±2, and 32±1 ng ABA per gram fresh weight (mean±SE), respectively. Drought stress caused a 19-, 24-, and 24-fold increase of ABA levels in wild-type, *nfxl2-1*, and *nfxl2-2* plants, respectively (ABA levels: 535, 780, and 751 ng ABA per gram fresh weight, Figure 9A). Thus, the *nfxl2-1* and *nfxl2-2* plants accumulated 46% and 40% more ABA under drought stress in comparison to the wild type.

Water stress also induces accumulation of reactive oxygen species (ROS) such as H_2_O_2_ [53], [54]. ROS can exacerbate cellular damage during stress, but also function as signalling compounds in plants. ROS modify the activity of protein kinases, transcription factors, and Ca^2+^ channels [55], [56]. Production of H_2_O_2_ was imaged in rosette leaves of drought-stressed plants. The *nfxl2-1* and *nfxl2-2* mutants displayed stronger staining in comparison to the wild type under mild drought stress conditions (Figure 9B, Figure S1). Thus, the loss of *NFXL2* enhanced H_2_O_2_ production. Water withdrawal for 5 d resulted in massive H_2_O_2_ production in all genotypes (Figure S1).

### The NFXL2-78 isoform largely complements the *nfxl2-1* mutant

Introduction of the *NFXL2-78* coding sequence under control of the CaMV35S promoter into the *nfxl2-1* mutant resulted in the specific expression of the *NFXL2-78* mRNA (Figure 2B). The *NFXL2-97* and *NFXL2-100* mRNAs were absent in the *nfxl2-1*/35S::NFXL2-78 lines. Stomatal aperture was increased in comparison to the mutant under favourable growth conditions (Figure 5A), stomatal conductance was increased to the wild-type level under drought stress (Figure 4), ABA-sensitivity of stomata, cotyledons, and roots was lower in comparison to the mutant (Figure 8), dry weight content (Table S1), survival rate after drought stress (Figure 3A), ABA-content (Figure 9A) and H_2_O_2_ levels (Figure 9B) were reduced to the wild-type level. Thus, the NFXL2-78 protein largely complemented the mutant phenotype.

Stable introduction of the *NFXL2-97* and *NFXL2-100* coding sequences into the *nfxl2-1* mutant (transgenic lines termed *nfxl2-1*/35S::NFXL2-97 and *nfxl2-1*/35S::NFXL2-100, respectively) resulted in the expression of the *NFXL2-97*/*NFXL2-78* and *NFXL2-100*/*NFXL2-97*/*NFXL2-78* mRNAs, respectively (Figure 2B). Thus, the *NFXL2-97* and *NFXL2-100* sequences allowed formation of the transcripts of two and three isoforms, respectively. This is a consequence of the presence of the corresponding donor and acceptor splice sites. The NFXL2-97 protein presumably can act both synergistically and antagonistically to NFXL2-78 in ABA responses. For example, simultaneous expression of the *NFXL2-78* and *NFXL2-97* mRNAs caused ABA-hyposensitivity of stomata, but ABA-hypersensitivity in the cotyledon greening assay (Figure S2, Figure S3). The *nfxl2-1*/35S::NFXL2-100 plants were hyposensitive to exogenous ABA (Figure S2, Figure S3). However, future analyses of the specific functions of the NFXL2-97 and NFXL2-100 isoforms will require mutagenesis of the donor and acceptor splice sites in the *NFXL2-97* and *NFXL2-100* coding sequences in order to allow specific expression of the single mRNAs.

## Discussion

### Structural features of NFXL2 protein isoforms

The NFXL2-100, NFXL2-97, and NFXL2-78 proteins are characterized by the major structural features that also characterize the human NFX1 protein, namely the Cys-rich region and the C_4_HC_3_ RING finger motif. This suggests common mechanisms of action. The human NFX1 protein is the only member of the NFX1/NFXL protein family that has been characterized at the molecular level. One isoform, NFXL1-91, was identified as a transcriptional repressor that binds to X-box elements at the *hTERT* promoter [34]. NFXL1-91 interacts with the corepressor mSin3A/HDAC to maintain the deacetylated status at the *hTERT* promoter [57]. Recently it was shown that NFX1-91 also binds to the p105 promoter and activates p105 expression [35]. Thus, NFX1-91 functions as a dual regulator, a transcriptional repressor, and also a transcriptional activator, when bound to DNA. The Arabidopsis NFXL2 proteins presumably also act as transcriptional regulators, because they form the Cys-rich region (Figure 1A) and are targeted to the nucleus (Figure 1B).

The RING finger motif of the NFX1/NFXL proteins suggests E3 ubiquitin ligase activity [36]. Nuclear E3 activity could control transcription or the destruction of proteins [41], [44]. However, in vivo targets of the NFX1/NFXL proteins are unknown, and the functional relevance of the RING finger has not been shown for any NFX1/NFXL protein. E3 activity of the NFXL2 proteins could not be tested so far due to insufficient amounts of recombinant protein (data not shown).

The human NFX1 and Arabidopsis NFXL2 proteins also display structural differences, which presumably result in functional divergence. The second analysed NFX1 isoform, NFX1-123, interacts with cytoplasmic poly(A) binding proteins (PABPs). Cytoplasmic PABPs stabilize RNA and promote translation. A site-directed mutagenesis approach demonstrated that the putative PAM2 motif [58] of the NFX1-123 isoform was critical to bind cytoplasmic PABPs [59]. However, the NFXL2 proteins lack a PAM2 motif, and also do not form other known PABP recognition motifs. Furthermore, the NFXL2 proteins do not contain the R3H domain which is present in NFX1 and many non-plant NFXL proteins. The R3H motif is supposed to mediate interactions with single stranded nucleic acids [60]. Thus, the NFXL2 proteins presumably do not play a role in the control of mRNA stability.

### *NFXL2* controls levels of ABA and hydrogen peroxide

The loss of *NFXL2* function caused an increase of approximately 10%ABA under favourable growth conditions (see above), and an increase of about 45% ABA after 3 d water withdrawal (Figure 9A). The increase of ABA is in line with phenotypic changes such as reduced transpiration (Figure 4A) and increased survival rates under water stress (Figure 3) and salt stress [31].

In addition to ABA, the *nfxl2-1* mutant also produced more hydrogen peroxide than the wild type under favourable growth conditions [31] and under mild drought stress conditions (Figure 9B, Figure S1). Water withdrawal for 5 d was accompanied by massive ROS production in all genotypes (Figure S1). ROS function as important signal transduction molecules. ABA-dependent stomata closure is partially dependent on hydrogen peroxide production [61]–[63]. ABA induces the activity of the plasma membrane NADPH oxidase [61], and H_2_O_2_ activates specific ion channels [64] and is essential for stomatal closing [62], [63].

Ascorbic acid is the major antioxidant that scavenges H_2_O_2_ [65]. Addition of ascorbate to epidermal peels or overexpression of dehydroascorbate reductase causes an increase in stomatal aperture and stomatal conductance [62], [63]. The elevated H_2_O_2_ levels in the *nfxl2-1* and *nfxl2-2* mutants may be a consequence of elevated ABA levels and presumably contribute to the reduced stomatal aperture. In line with this interpretation, application of 10 mM ascorbate significantly reduced ABA-induced closing of *nfxl2-1* stomata (151% aperture in comparison to ABA alone), whereas a minor effect was observed in the wild type (110% aperture) (Figure S4).

### *NFXL2* suppresses ABA responses

Responses to exogenous ABA were enhanced in the *nfxl2-1* mutant (stomatal closure: Figure 8A,C; inhibition of cotyledon greening: Figure 8D; and inhibition of root growth: Figure 8E). Introduction of the NFXL2-78 isoform largely complemented the *nfxl2-1* mutant phenotypes. The NFXL2-100 isoform may play a similar role as the NFXL2-78 protein, but action of the NFXL2-97 protein could be antagonistic in a subset of ABA responses (Figure S2, Figure S3).

*NFXL2* gene products suppress both ABA accumulation (Figure 9A) and ABA responses (Figure 8). The molecular basis of this dual role is unknown, and different models are conceivable. Experimental evidence indicates that *NFXL2* could control ABA levels and ABA responses via transcriptional control of the plant clock. *NFXL2* (also termed *EARLY BIRD* [*EBI*]) was shown to associate with ZEITLUPE (ZTL) and to regulate the expression of components of the circadian clock [66]. A large number of ABA-responsive genes oscillate diurnally [67], and ABA metabolic genes are also clock-regulated [68]. Clock-dependent circadian modulation (‘gating’) of ABA function is important for cellular homeostasis under dehydration stress [69]–[71]. Alternatively, *NFXL2* could control the level or activity of other nuclear factors involved in the control of ABA responses, or *NFXL2* could control regulatory mechanisms upstream of ABA signalling (e.g. desensitize osmosensors or repress signalling events downstream of the osmosensors).

*NFXL1* and *NFXL2* supposedly play antagonistic roles in the control of abiotic stress responses. *NFXL1* promotes the adaptation to salt stress [31] and drought stress (data not shown), *NFXL2* suppresses the adaptation to salt stress [31] and drought stress (Fig. 3). Future work will address the specific roles of the NFXL2 proteins, the interplay between the NFXL2 isoforms, and the interplay between the *NFXL1* and *NFXL2* genes.

## Materials and Methods

### Screen for mutants and establishment of transgenic lines

The GABI-Kat line 668B09 [72] carries a T-DNA insertion in an *NFXL2* intron (Figure 1A) and was named *nfxl2-2*. The DNA insertion site was confirmed by sequencing. Homozygosity of T-DNA insertions was confirmed by PCR on genomic DNA using T-DNA border-specific and gene-specific primers. The *NFXL2-78*, *NFXL2-97*, and *NFXL2-100* coding sequences were amplified using the primers NFXL2-78/97/100-fw 5’ CAC CAT GAC TAA TAT GGC CGG AAC CG 3’, NFXL2-78-rev 5’ TTA ACA CCG ATT CAG CCA CCT GTA G 3’, and NFXL2-97/100-rev 5’ TTA GAT TCG AGG GTA TCT TCT AGA C 3’. The PCR fragments were cloned into the pENTR/D-TOPO (Invitrogen, Karlsruhe, Germany) vector, and used to establish GFP fusion constructs using the pK7FWG2 vector [73]. The *NFXL2-78*, *NFXL2-97*, and *NFXL2-100* sequences were also inserted into the pH7WG2 vector for expression under control of the 35S promoter [73]. Sequence analysis of all cloned PCR products revealed 100% identity to the respective cDNA sequence. All constructs were transformed into Arabidopsis plants using the floral-dip method.

### Growth conditions

Plants were established in soil. Seeds were allowed to germinate and grew for two weeks in controlled growth chambers (7 days: 16 h light, 140 µmol m^−2^ s^−1^, 20°C, 75% relative humidity; 8 h night, 6°C, 75% relative humidity; thereafter 7 days: 8 h light, 140 µmol m^−2^ s^−1^, 20°C, 60% relative humidity; 16 h night, 16°C, 75% relative humidity). Subsequently, plants were transferred to long day conditions in a greenhouse with artificial light (16 h light [high pressure sodium and metal halide lamps], 21°C, 50% relative humidity; 8 h night, 19°C, 50 % relative humidity) or to specific conditions as indicated in the text. All genotypes were grown side by side in a randomized manner. Alternatively, plants were grown under aseptic conditions as described before [31]. For ABA induction, a few droplets of 10 or 100 µM ABA (Duchefa, Haarlem, The Netherlands) dissolved in stomata opening buffer (SOB: 5 mM MES, 10 mM KCl, 50 µM CaCl_2_, pH 6.15) were added to the petioles of fully expanded rosette leaves.

### Gene expression analysis

The *NFXL2* transcripts could not be detected by means of Northern-blot analysis and were analyzed by RT-PCR as described [31]. Sequences of primers used for RT-PCR analysis were as follows: NFXL2-78-fw 5’ AAG GCG CGC TCC TCC CTT GT 3’, NFXL2-78-rev 5’ CAC CGA TTC AGC CAC CTG TA 3’, NFXL2-97-fw 5’ CCG TGG ACC TTG TCA CAG AAA 3’, NFXL2-97-rev 5’ CGA ACA ACC ACC TTT TTA CCA CA 3’, NFXL2-100 fw 5’ ATA TAT CCA TTT GGG ATG CTG TAT CT 3’, and NFXL2-100-rev 5’ GCA GCT AGC ATC GCC ACT AA 3’. The *eIF1α*, *PDF1*, and *LRS1* genes were used to normalize the expression levels. Sequences of primers were as follows: eIF1*α*-fw 5’ TTG ACA GGC GTT CTG GTA AGG 3’, eIF1*α*-rev 5’ CAG CGT CAC CAT TCT TCA AAA A 3’, PDF1-fw 5’ ACG TCG CTA AAG TAC TTC AAT CCC 3’, PDF1-rev 5’ CGA ATC GTC TTC TCC ACA ACC G 3’, LRS1-fw 5’ ATG GGC ATT TGA CGA GGA TGC G3’, and LRS1-rev 5’ CGT CGT TCA CCC AGT CAA CAT GAG 3’. The amplification efficiency of each PCR was computed with the LinRegPCR software [74] and was used to correct the readout for each primer pair and run.

### Measurement of ABA levels and DAB staining

ABA extraction was done at 4°C with dimmed light. 50 mg powdered tissue were subjected to lyophilisation for 24 h. Tissue was suspended in 0.5 ml extraction buffer (MeOH containing 2.5 mM citric acid monohydrate and 0.5 mM butylated hydroxytoluene). The extract was incubated for at least 20 h in the dark at 4°C under shaking conditions and centrifuged at 1500×g for 15 min at 4°C. The supernatants were recovered and 2 ml 62.5% extraction buffer/28.5% MeOH was added. C18 Sep-Pak cartridges (Waters, Eschborn, Germany) were equilibrated with 2 ml extraction buffer and subsequently with 1 ml 70% extraction buffer/30% MeOH. Supernatants were passed through a C18 Sep-Pak cartridge. 1 ml 70% MeOH was loaded onto the cartridge and flow-throughs were united. Additional elutions were analyzed exemplarily to check for residual ABA on the cartridge. The eluates were dried in a lyophilizer. Dried samples were resuspended in 1 ml 50 mM Tris buffered saline pH 7.5 (TBS)/MeOH (10 :1). Dilutions in TBS of each sample extract or ABA standard (Duchefa) were subjected to analysis by an enzyme immunoassay using the PGR1 kit (Sigma-Aldrich) according to the manufacturer' instruction. The ABA standard was assumed to be a mixture of equal amounts of the (S)-2-cis and (S)-2-trans form of ABA.

The production of H_2_O_2_ was imaged in plant material infiltrated with 3,3-diaminobenzidine (DAB) [75]. A method described in [76] was used for quantitative H_2_O_2_ measurements.

### Microscopy

GFP-fluorescence was visualized in roots of 14-day-old transgenic plants grown in sterile media using a Leica TCS SP5 confocal microscope.

### Stomatal aperture, stomatal conductance, leaf temperature, and gas exchange measurements

Stomatal aperture width was determined by light microscopy of nail polish images from silicone rubber imprints of abaxial surfaces of rosette leaves as described in [77]. Alternatively, epidermal peels were prepared from 5-week-old plants grown in a greenhouse, mounted on a microscope slide, and analyzed by light microscopy. Stomatal conductance of rosette leaves was determined abaxially using the Decagon SC-1 porometer after adjusting the clamp pressure of the sensor head to measurements with Arabidopsis according to the manufacturer’s instructions. Infrared gas exchange measurements were performed as described before [78]. 10 µM fusicoccin (Sigma-Aldrich) was added to SOB and applied to epidermal peels. Leaf temperature was measured by an infrared thermal imaging camera (PIR uc 180 InfraTec, Dresden, Germany). Images were recorded online by application of the IRBIS-3 thermography software (InfraTec, Dresden, Germany).

## Supporting Information

Figure S1 **Hydrogen peroxide levels.** A. For drought treatment, soil-grown plants were withheld from water for 5 d. Rosette leaves were infiltrated with 3,3-diamino-benzidine (DAB). Formation of brown polymerisation product indicates H_2_O_2_ formation. B. For drought treatment, soil-grown plants were withheld from water for 1, 3, and 5 d. H_2_O_2_ levels were determined as described in [76].
(PDF)

Figure S2 **Stomatal aperture after ABA application.** For details see Fig. 8A.
(PDF)

Figure S3 **Cotyledon greening in the presence of exogenous ABA.** For experimental details see Fig. 8D.
(PDF)

Figure S4 **Stomatal aperture in the presence of ABA and ascorbate.** Epidermal peels were dissected from rosette leaves of soil-grown plants and incubated for 5 h in light (120 µmol m^−2^ s^−1^) either in stomata opening buffer supplemented with 10 µM ABA or in stomata opening buffer supplemented with 10 µM ABA+10 mM ascorbate. Aperture of untreated stomata is shown as reference. Aperture of ABA+ascorbate treated *nfxl2-1* stomata is significantly different from ABA treated stomata (t test, P<0.001). Percent difference (ABA+ascorbate vs. ABA) was significantly larger in *nfxl2-1* in comparison to the wild type (t test, P<0.001).
(PDF)

Table S1 **Dry weight content.**
(PDF)
