# Supplementary figures and images for: NFX1-LIKE2 (NFXL2) Suppresses Abscisic Acid Accumulation and Stomatal Closure in Arabidopsis thaliana
Source: PLoS One. 2011 Nov 3;6(11):e26982. doi: 10.1371/journal.pone.0026982 (PMC3207813; doi:10.1371/journal.pone.0026982)

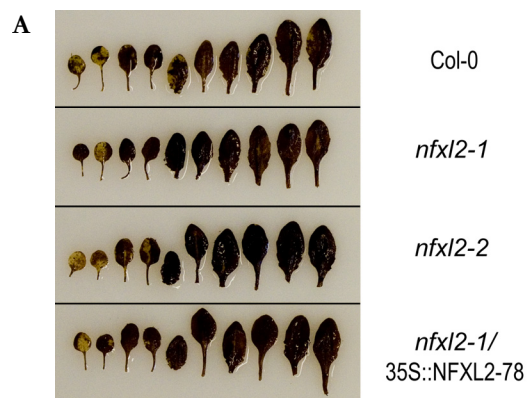

**Figure S1. Hydrogen peroxide levels.**

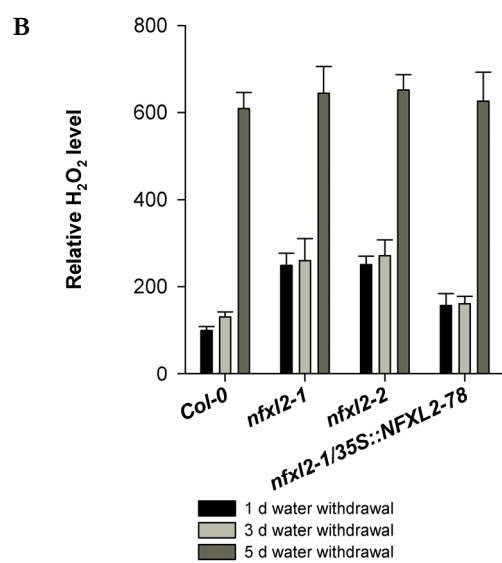

Supplement: Figure S1 — Hydrogen peroxide levels. A. For drought treatment, soil-grown plants were withheld from water for 5 d. Rosette leaves were infiltrated with 3,3-diamino-benzidine (DAB). Formation of brown polymerisation product indicates H2O2 formation. B. For drought treatment, soil-grown plants were withheld from water for 1, 3, and 5 d. H2O2 levels were determined as described in [76]. (PDF) [file pone.0026982.s001.pdf]

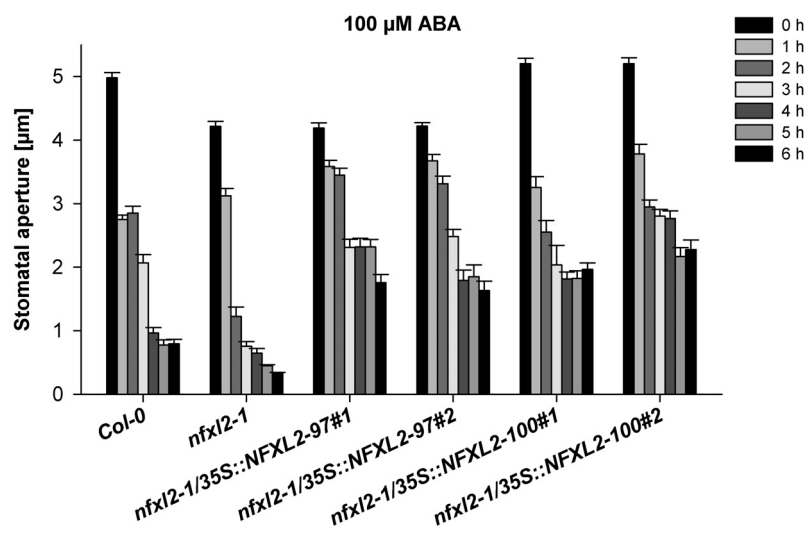

Figure S2. Stomatal aperture after ABA application.

Supplement: Figure S2 — Stomatal aperture after ABA application. For details see Fig. 8A. (PDF) [file pone.0026982.s002.pdf]

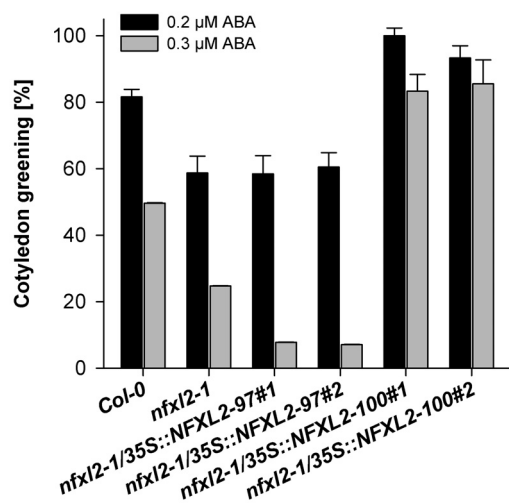

**Figure S3.** Cotyledon greening in the presence of exogenous ABA.

Supplement: Figure S3 — Cotyledon greening in the presence of exogenous ABA. For experimental details see Fig. 8D. (PDF) [file pone.0026982.s003.pdf]

Figure S4. Stomatal response to ABA and ascorbate.

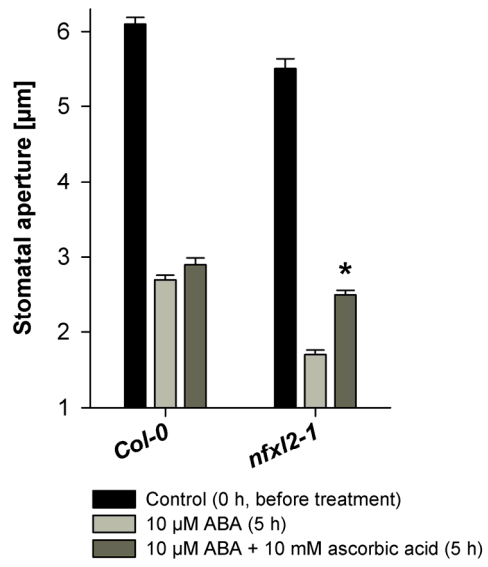

Supplement: Figure S4 — Stomatal aperture in the presence of ABA and ascorbate. Epidermal peels were dissected from rosette leaves of soil-grown plants and incubated for 5 h in light (120 µmol m−2 s−1) either in stomata opening buffer supplemented with 10 µM ABA or in stomata opening buffer supplemented with 10 µM ABA+10 mM ascorbate. Aperture of untreated stomata is shown as reference. Aperture of ABA+ascorbate treated nfxl2-1 stomata is significantly different from ABA treated stomata (t test, P<0.001). Percent difference (ABA+ascorbate vs. ABA) was significantly larger in nfxl2-1 in comparison to the wild type (t test, P<0.001). (PDF) [file pone.0026982.s004.pdf]
