# Supplementary material for: NFX1-LIKE2 (NFXL2) Suppresses Abscisic Acid Accumulation and Stomatal Closure in Arabidopsis thaliana
Source: PLoS One. 2011 Nov 3;6(11):e26982. doi: 10.1371/journal.pone.0026982 (PMC3207813; doi:10.1371/journal.pone.0026982)
Supplement: Table S1 — Dry weight content. (PDF) [file pone.0026982.s005.pdf]

Table S1. Dry weight content (in % fresh weight). Mean and SE of four independent experiments.

Well-watered four-week-old plants (long day standard conditions)

	**Col-0**	***nfxl2-1***	***nfxl2-2***	***nfxl2-1/* 35S::NFXL2-78#1**	***nfxl2-1/* 35S::NFXL2-78#2**
**mean**	8.23	9.28	9.92	7.92	8.04
**% Col-0**		112	121	96	98
**SE**	0.35	0.42	0.66	0.40	0.57
**t test vs. Col-0**		0.008	0.007	0.761	0.683

Drought-stressed plants (long day standard conditions, water withdrawal for 3 days)

	**Col-0**	***nfxl2-1***	***nfxl2-2***	***nfxl2-1/* 35S::NFXL2-78#1**	***nfxl2-1/* 35S::NFXL2-78#2**
**mean**	8.48	9.78	10.42	8.84	8.25
**% Col-0**		115	123	102	104
**SE**	0.36	0.29	0.50	1.35	1.00
**t test vs. Col-0**		0.007	0.007	0.798	0.721
